# The mechanism of TGF-β/miR-155/c-Ski regulates endothelial–mesenchymal transition in human coronary artery endothelial cells

**DOI:** 10.1042/BSR20160603

**Published:** 2017-08-24

**Authors:** Juan Wang, Wen He, Xiao Xu, Liping Guo, Yin Zhang, Suxia Han, Difei Shen

**Affiliations:** 1 Department of Cardiology, The Fifth Affiliated Hospital of Xinjiang Medical University, Urumchi 830001, Xinjiang, China; 2 Department of Pharmacy, The Fifth Affiliated Hospital of Xinjiang Medical University, Urumchi 830001, Xinjiang, China; 3 Department of Oncology, The First Affiliated Hospital of Xinjiang Medical University, Urumchi 830001, Xinjiang, China; 4 Department of Cardiology, Renmin Hospital of Wuhan University, Wuhan 430060 Hubei, China

**Keywords:** cardiac fibrosis, c-Ski, endothelial-mesenchymal transition (EndMT), Human coronary artery endothelial cells (HCAECs), miR-155, transforming growth factor-β (TGF-β)

## Abstract

Human coronary artery endothelial cells (HCAECs) have the potential to undergo fibrogenic endothelial–mesenchymal transition (EndMT), which results in matrix-producing fibroblasts and thereby contributes to the pathogenesis of cardiac fibrosis. Recently, the profibrotic cytokine transforming growth factor-β (TGF-β) is shown to be the crucial pathogenic driver which has been verified to induce EndMT. C-Ski is an important regulator of TGF-β signaling. However, the detailed role of c-Ski and the molecular mechanisms by which c-Ski affects TGF-β-induced EndMT in HCAECs are not largely elucidated. In the present study, we treated HCAECs with TGF-β of different concentrations to induce EndMT. We found that overexpression of c-Ski in HCAECs either blocked EndMT via hindering Vimentin, Snail, Slug, and Twist expression while enhancing CD31 expression, with or without TGF-β treatment. In contrast, suppression of c-Ski further enhanced EndMT. Currently, miRNA expression disorder has been frequently reported associating with cardiac fibrosis. By using online tools, we regarded miR-155 as a candidate miRNA that could target c-Ski, which was verified using luciferase assays. C-Ski expression was negatively regulated by miR-155. TGF-β-induced EndMT was inhibited by miR-155 silence; the effect of TGF-β on Vimentin, CD31, Snail, Slug, and Twist could be partially restored by miR-155. Altogether, these findings will shed light on the role and mechanism by which miR-155 regulates TGF-β-induced HCAECs EndMT via c-Ski to affect cardiac fibrosis, and miR-155/c-Ski may represent novel biomarkers and therapeutic targets in the treatment of cardiac fibrosis.

## Introduction

Fibrosis has recently been reported to be linked with a decreased extent of microvasculature and disrupted normal myocardial structures, as a result of excessive deposition of extracellular cell matrix mediated by recruitment of fibroblasts and endothelial dysfunction, but not much is known about its contribution to these conditions [1].

Endothelial–mesenchymal transition (EndMT) is identified as a complex biological process during which endothelial cells lose various particular markers and obtain mesenchymal or myofibroblastic phenotype and consequently express mesenchymal cell products [2]. EndMT is a key event in cancer metastasis, which confers cancer cells with increased motility and invasiveness, and is characterized by loss of endothelial marker CD31 and gain of mesenchymal marker Vimentin [2,3]. According to previous studies, EndMT process is closely associated with renal, lung, or cardiac fibrosis [4,5]. Zeisberg et al. [6] first revealed that cardiac fibrosis had notable association with the appearance of fibroblasts originating from endothelial cells, which suggested an analogous event to EndMT that occurs during formation of the atrioventricular cushion in the embryonic heart. Then, it has also been reported that endothelin-1 derived from endothelial cell-promoted cardiac fibrosis via EndMT [7]. However, the mechanism of EndMT in human coronary artery endothelial cells (HCAECs) remains to be investigated.

During this process, the transforming growth factor-β (TGF-β)/Smad pathway functioned as a key promoter [8,9]. Up-regulated glomerular TGF-β expression has been reported in both experimental and human kidney disease [1,10,11]. Mice with increased plasma TGF-β1 levels sustained promoted cardiac fibrosis [12]. TGF-β signaling promotes fibroblast survival and proliferation by inducing target genes, or regulated by several factors including c-Ski [13]. TGF-β induced a rapid- and dose-dependent degradation of c-Ski protein in human melanoma cells [14]; c-Ski inhibits TGF-β signaling through interaction with Smad proteins [13].

The entire range of TGF-β-target genes includes microRNAs (miRNAs) [15]. miRNAs are small (21–23 nt) non-coding RNA molecules involved in gene expression by interacting with diverse mRNAs and inducing either translational suppression or mRNA degradation [16]. MiRNAs are involved in regulating multiple physiological processes including embryogenesis, organ development, oncogenesis, and the earliest step in EndMT [17–20]. In addition, cardiac disease and development are substantially regulated by miRNAs [21]. Therefore, miRNAs have been suggested as clinically relevant targets of cardiac disease [22].

In the present study, we found that overexpression of c-Ski in HCAECs blocked EndMT via hindering Vimentin, Snail, Slug, and Twist expression while enhancing CD31 expression, with or without TGF-β treatment. MiR-155 was identified as a candidate miRNA that could target c-Ski, which was verified using luciferase assays. MiR-155 expression was negatively regulated by c-Ski. TGF-β-induced EndMT was inhibited by miR-155 silence; the effect of TGF-β on Vimentin and CD31 could be partially restored by miR-155. Altogether, these findings will shed light on the role and mechanism by which miR-155 regulates TGF-β-induced HCAECs EndMT via c-Ski to affect cardiac fibrosis, and miR-155/c-Ski may represent novel biomarkers and therapeutic targets for cardiac fibrosis.

## Materials and methods

### Cell lines and treatment

We purchased HCAEC cell lines from Lonza (Basel, Switzerland) and maintained them in DMEM/F12 medium (Invitrogen, CA, U.S.A.) supplemented with 10% fetal bovine serum (Gibco, CA, U.S.A.) and 100 U/ml penicillin and 100 µg/ml streptomycin in a humidified 5% CO_2_, 95% O_2_ incubator at 37°C. Cells were treated with different concentration of TGF-β (0, 1, 2.5, and 5 ng/ml) for 48 h. The morphology change was observed by a microscope (Olympus, Japan). Total proteins were extracted for further experiments.

### Cell transfection

HCAECs were plated in six-well plates and cultured for 24 h. By transfection with miR-155 mimics or miR-155 inhibitor (Genepharma, Shanghai, China) (a final concentration of 50 nM) using Lipofectamine 2000 (Invitrogen, CA, U.S.A.) for 48 h, cells were achieved ectopic expression or inhibition of miR-155. By transfection with 2 μg of PcDNA3.1/c-Ski or sh-c-Ski vector (GeneCopoecia, Guangzhou, China) using Lipofectamine 2000 for 48 h, cells were achieved overexpression or knockdown of c-Ski. Total RNAs and proteins were extracted after 48 h of transfection.

### RNA extraction and SYBR green quantitative real-time PCR

Trizol reagent as recommended by the manufacturer was used to extract total RNA (Invitrogen, CA, U.S.A.). A NanoDrop 2000c instrument was used to evaluate RNA quality and concentration (ThermoScientific, MA, U.S.A.). A Hairpin-it™ miRNAs qPCR Quantitation Kit (Gene Pharma, Shanghai, China) was used to detect mature miR-155 for miRNA analysis. U6 was used as an endogenous control. We performed real-time PCR using PowerSYBR green PCR master mix to analyze c-Ski mRNA (Takara, Dalian, China) on an ABI 7900HCAECPCR machine. GAPDH was used for normalization for applied Biosystems and data unless otherwise indicated. The 2^−ΔΔ*C*^_t_ method was used for data analysis.

### Western blot

Cell lysates were lysed by RIPA buffer (Sigma-Aldrich, U.S.A.) with Complete Protease Inhibitor Cocktail (Roche, U.S.A.). Cell lysates were transferred to 1.5 ml tube and kept at −20°C before use. SDS/PAGE was conducted to separate the cellular proteins, and all the cellular proteins within the study were separated by 5% stacking gel and 10% running gel. The molecular weight of candidate proteins was referred to the information of the Pre-stained SeeBlue rainbow marker (Invitrogen, U.S.A.) loaded in parallel. The membranes were probed with the following antibodies: c-Ski, CD31, Vimentin, Snail, Slug, and Twist (Abcam, MA, U.S.A.), and GAPDH (Sigma, U.S.A.). The blots were detected on Kodak film developer (Fujifilm, Japan).

### C-Ski 3′-UTR Luciferase reporter assays

HCAECs were seeded into a 24-well plate. After cultured overnight, cells were co-transfected with the wild-type or mutated c-Ski 3′-UTR reporter plasmid and miR-155 mimics or miR-155 inhibitor. Luciferase assays were performed 48 h after transfection using the Dual Luciferase Reporter Assay System (Promega, WI, U.S.A.).

### Statistical analysis

Values are shown as means ± s.d of three independent experiments unless otherwise specified. SPSS17.0 (Pearson) was used to analyze data by unpaired student *t*-test or by ANOVA. *P*<0.05 was considered to be statistically significant.

## Results

### TGF-β induces EndMT in HCAECs

HCAECs were treated with a series of doses of TGF-β for 48 h, and then the cellular morphology, expression trend of endothelial marker CD31, and mesenchymal maker Vimentin, Snail, Slug, and Twist were monitored to verify the induction of EndMT by TGF-β. Cell morphology was photographed using a phase-contrast microscope as exhibited in Figure 1(A). Following TGF-β stimulation, HCAECs exhibited a spindle-shaped and fibroblast-like morphology and exhibited an EndMT phenotype. Notably, along with the increase of TGF-β concentration, the EndMT phenotype of HCAECs exhibited more obviously (Figure 1A). We further verified the inductive effect of TGF-β on EndMT in HCAECs by determining the protein expression of CD31, Vimentin Snail, Slug, and Twist. As exhibited using Western blot assays, CD31 protein expression was significantly down-regulated, while Vimentin, Snail, Slug, and Twist protein expression were significantly up-regulated by TGF-β in a dose-dependent manner (Figure 1B–G). These data suggested that TGF-β successfully induced EndMT in HCAECs.

**Figure 1:**
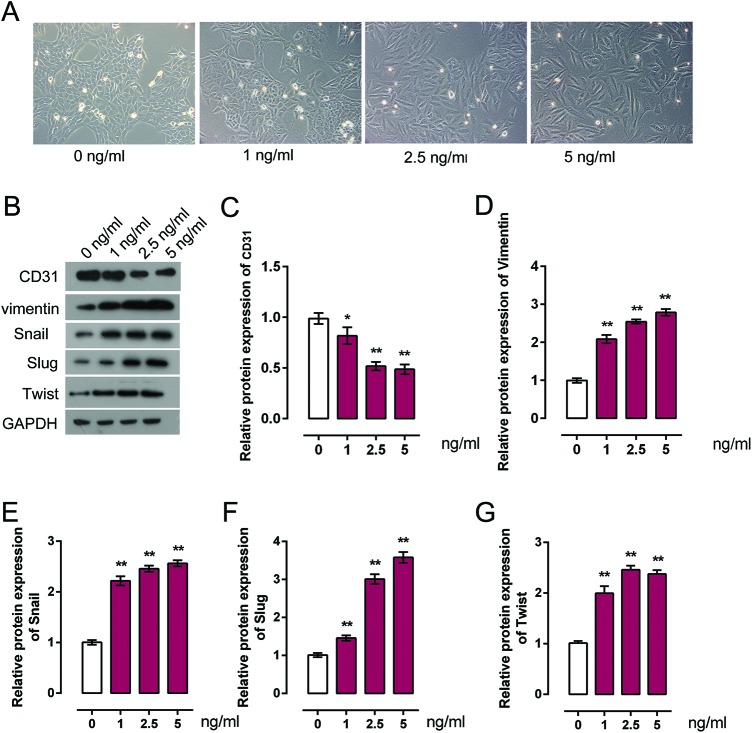
TGF-β induces EndMT in HCAECs (**A**) HCAECs were treated with TGF-β (0, 1, 2.5, and 5 ng/ml), and the cell morphology was photographed using a phase-contrast microscope. (**B**–**G**) HCAECs were treated with TGF-β (0, 1, 2.5, and 5 ng/ml), the protein expression of CD31, Vimentin, Snail, Slug, and Twist after TGF-β treatment was monitored in HCAECs using Western blot. Results from the Western blot assays were then analyzed using gradation analysis. The data were analyzed by ANOVA and presented as mean ± s.d of three independent experiments.

### Detailed role of c-Ski in TGF-β-induced EndMT of HCAECs

Downregulation of c-Ski was reported to be involved in enhanced proliferation as it is a characteristic in cancer or fibrosis [23,24]. Actually, a recent study confirmed c-Ski function as regulator of kidney fibrosis [23]. We further explored whether c-Ski contribute to TGF-β-induced EndMT. HCAECs were treated with a series of doses of TGF-β (0, 1, 2.5, and 5 ng/ml), and then c-Ski protein expression was monitored using Western blot. As exhibited in Figure 2, the protein expression of c-Ski was promoted under TGF-β treatment, and the most obvious change in expression trend occurred under 5 ng/ml TGF-β treatment (Figure 2A). Next, pc3.1DNA/c-Ski or sh-c-Ski was transfected into HCAECs to achieve c-Ski overexpression or knockdown. Transfection efficiency was verified using Western blot. Results showed that the protein expression of c-Ski was significantly up-regulated or inhibited by pc3.1DNA/c-Ski or sh-c-Ski transfection (Figure 2B). Then we monitored the expression changes of EndMT markers, CD31, Vimentin, Snail, Slug, and Twist using Western blot. Results showed that 5 ng/ml TGF-β treatment significantly reduced CD31 protein expression while promoted Vimentin, Snail, Slug, and Twist protein expression; c-Ski overexpression significantly promoted CD31 protein expression while reduced Vimentin, Snail, Slug, and Twist expression; c-Ski overexpression could partially restore the effect of TGF-β on CD31, Vimentin, Snail, Slug, and Twist (Figure 2C–H). In contrast, c-Ski knockdown significantly inhibited CD31 protein expression while increased Vimentin, Snail, Slug, and Twist expression; c-Ski knockdown could enhance the effect of TGF-β on CD31, Vimentin, Snail, Slug, and Twist (Figure 3B–F). These data revealed that c-Ski could hinder TGF-β-induced EndMT by promoting CD31 protein expression and reducing Vimentin, Snail, Slug, and Twist protein expression.

**Figure 2:**
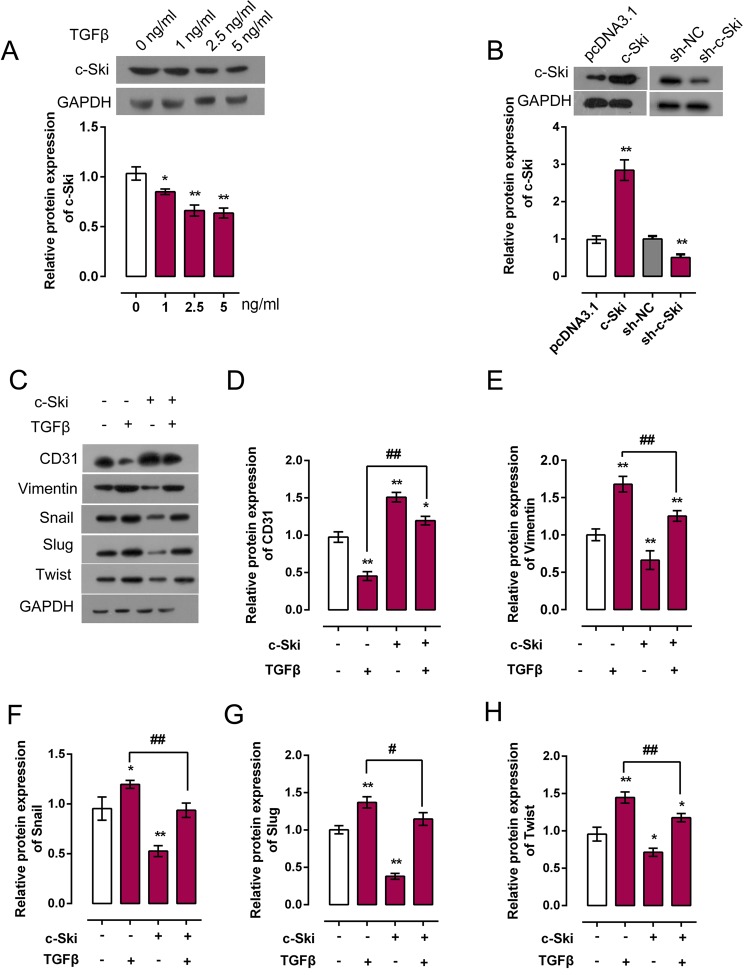
Detailed role of c-Ski in TGF-β-induced EndMT of HCAECs (**A**) HCAECs were treated with TGF-β (0, 1, 2.5, and 5 ng/ml), the protein expression of c-Ski under different concentrations of TGF-β was monitored using Western blot assays. (**B**) PcDNA3.1/c-Ski or Sh-c-Ski vector was transfected into HCAECs to achieve c-Ski overexpression or knockdown. Transfection efficiency was verified using Western blot assays. (**C**–**H**) PcDNA3.1/c-Ski was transfected into HCAECs with or without 5 ng/ml TGF-β treatment; the protein expression of EndMT markers CD31, Vimentin, Snail, Slug, and Twist was monitored using Western blot assays. Results from Western blot assays were then analyzed using gradation analysis. The data were analyzed by student *t*-test or ANOVA and presented as mean ± s.d of three independent experiments.

**Figure 3:**
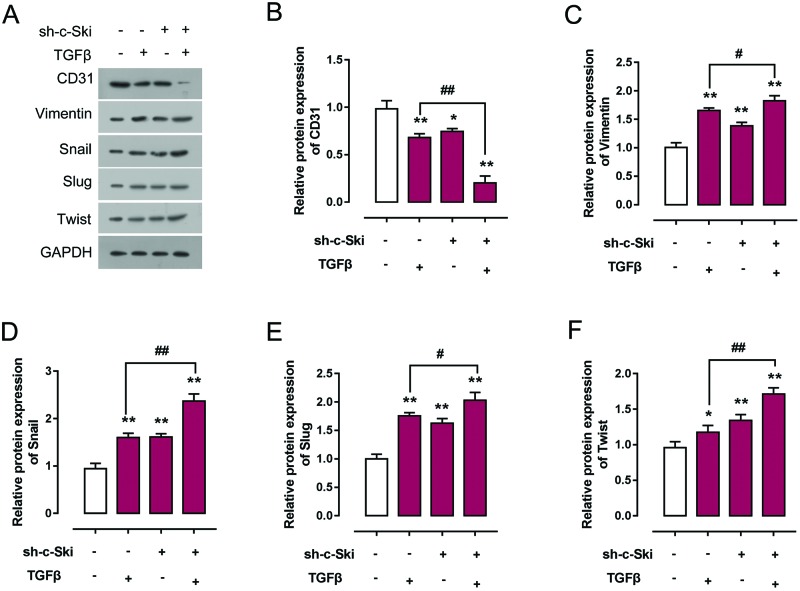
Knockdown of c-Ski promotes TGF-β-induced EndMT of HCAECs (**A**–**F**) Sh-c-Ski was transfected into HCAECs with or without 5 ng/ml TGF-β treatment; the protein expression of EndMT markers CD31, Vimentin, Snail, Slug, and Twist was monitored using Western blot assays. Results from Western blot assays were then analyzed using gradation analysis. The data were analyzed by student *t*-test or ANOVA and presented as mean ± s.d of three independent experiments.

### MiR-155 regulates c-Ski expression via direct binding to c-Ski 3′-UTR

A series of independent scientific research has revealed that miRNAs are involved in the regulation of EndMT and cardiac disease [20,21]. We speculated that miRNAs may impact the process of TGF-β-induced EndMT. To confirm this speculation, online tools including miRWalk, miRanda, TargetScan, and DIANA were using to scan out the candidate miRNAs for c-Ski regulation. Combined with previous studies, we chose seven candidate miRNAs which have been reported to be regulated by TGF-β treatment: miR-21, miR-155, miR-367, miR-497, miR-15a, miR-195, and miR-221 [25–29] (Figure 4A). MiRNA mimics were transfected into HCAECs, and the expression efficiency was verified using real-time PCR (Figure 4B). Results showed that the expression of all the seven miRNAs were significantly up-regulated by miRNA mimics respectively (Figure 4B).To confirm the regulation of c-Ski by miRNAs, mRNA expression of c-Ski in response to miRNAs’ overexpression was monitored using real-time PCR. Results showed that c-Ski expression could be down-regulated by all the seven miRNAs, of which miR-155 exhibited the highest inhibitory efficiency (Figure 4C). Given all these, miR-155 was selected as the study subject. MiR-155 inhibitor was then transfected into HCAECs to achieve miR-155 inhibition, and the inhibitory efficiency was verified using real-time PCR (Figure 4D). C-Ski protein expression in response to miR-155 overexpression or inhibition was monitored using Western blot. Results showed that c-Ski protein expression was negatively regulated by miR-155: miR-155 overexpression down-regulated c-Ski protein expression, while miR-155 inhibition up-regulated c-Ski protein expression (Figure 4E). Since miR-155 and c-Ski 3′-UTR have the same seed sequence as shown, we next explored whether miR-155 directly binds to c-Ski on translational repression. Luciferase reporter vectors incorporating a wild-type or mutant 3′-UTR of c-Ski in which the sequence that corresponds to the seed region has been altered were constructed (Figure 4F). Co-transfection of wild-type c-Ski luciferase reporter vectors with miR-155 mimics in HCAECs lead to an observably attenuated c-Ski 3′-UTR luciferase activity expression, while co-transfection of wt-c-Ski with miR-155 inhibitor lead to a promoted c-Ski 3′-UTR luciferase activity expression. When it came to the mut-c-Ski luciferase reporter vector, the co-transfection with either miR-155 mimics or miR-155 inhibitor lead to no significant change of luciferase activity, which confirmed that miR-155 directly suppressed luciferase activity together with the wild-type 3′-UTR of c-Ski (Figure 4G), but not with the mutant version of c-Ski. Altogether, miR-155 exerted its inhibitory function via direct targeting 3′-UTR of c-Ski.

**Figure 4:**
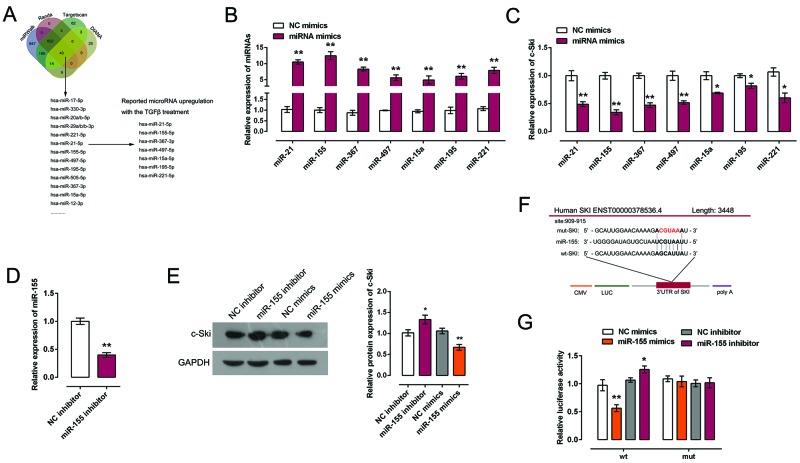
MiR-155 regulates c-Ski expression via direct binding to c-Ski 3′-UTR (**A**) Online tools including miRWalk, miRanda, TargetScan, and DIANA were using to scan out the candidate miRNAs for c-Ski regulation. Seven of them were reported by previous studies that could be regulated by TGF-β. (**B**) MiRNA mimics of the seven candidate miRNAs were transfected into HCAECs respectively. Transfection efficiency was verified using real-time PCR. (**C**) MiRNA mimics of the seven candidate miRNAs were transfected into HCAECs respectively. Expression of c-Ski in response to overexpression of indicated miRNAs was monitored using real-time PCR. (**D**) MiR-155 inhibitor was transfected into HCAECs to achieve miR-155 inhibition. Inhibitory efficiency was verified using real-time PCR. (**E**) MiR-155 mimics or miR-155 inhibitor was transfected into HCAECs respectively; the protein expression of c-Ski was monitored in response to miR-155 overexpression or inhibition using Western blot assays. (**F**) Luciferase reporter gene vector containing a wild-type c-Ski 3′-UTR or mutant c-Ski 3′-UTR by mutating 5 bp on the predicted binding site of miR-155 in c-Ski 3′-UTR. (**G**) wt-c-Ski or mut-c-Ski was co-transfected into HCAECs with miR-155 mimics or miR-155 inhibitor. Changes of luciferase activity of c-Ski 3’-UTR reporter gene vector were monitored. The data were analyzed by student *t*-test or ANOVA and presented as mean ± s.d of three independent experiments.

### The detailed role of miR-155 in TGF-β-induced EndMT

Since we have revealed that miR-155 regulates c-Ski expression by direct targeting its 3′-UTR, and that c-Ski plays a role in TGF-β-induced EndMT, we further investigated the detailed function of miR-155 in TGF-β-induced EndMT. MiR-155 expression in response to TGF-β treatment was monitored using real-time PCR. Results showed that TGF-β treatment significantly induced miR-155 expression (Figure 5A). Then the protein expression of EndMT markers, CD31, Vimentin, Snail, Slug, and Twist was monitored in response to miR-155 inhibition with or without TGF-β treatment. Results showed that miR-155 inhibition could significantly promote CD31 protein expression while reduce Vimentin, Snail, Slug, and Twist protein expression; the effect of TGF-β on CD31, Vimentin, Snail, Slug, and Twist could be partially restored by miR-155 inhibition (Figure 5B–G). These data showed that miR-155 inhibition could hinder TGF-β-induced EndMT by promoting CD31 expression and reducing Vimentin Snail, Slug, and Twist expression; the effect of TGF-β on EndMT markers could be partially restored by miR-155 inhibition.

**Figure 5:**
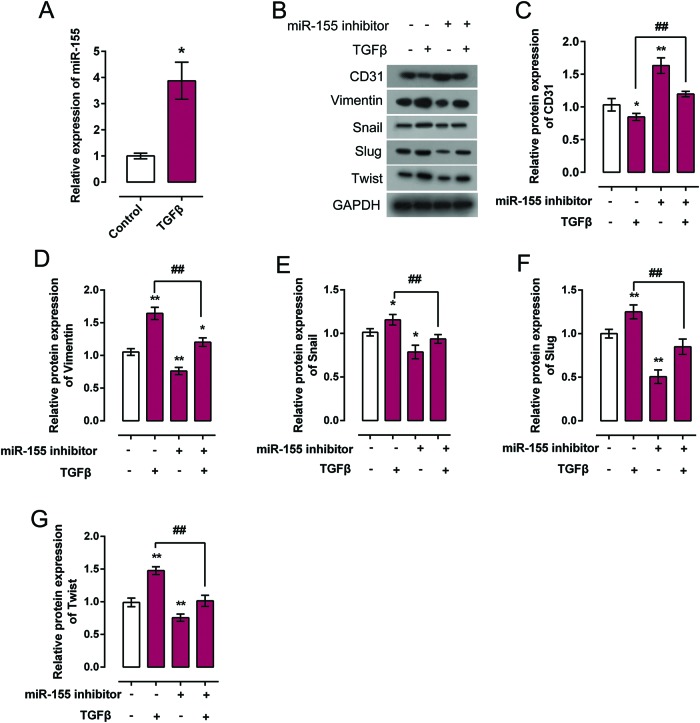
The detailed role of miR-155 in TGF-β-induced EndMT (**A**) miR-155 expression in response to 5 ng/ml TGF-β treatment was monitored using real-time PCR in HCAECs. (**B–G**) Protein expression of EndMT markers CD31, Vimentin, Snail, Slug, and Twist in response to miR-155 overexpression or inhibition with or without TGF-β treatment was monitored using Western blot. Results from Western blot were analyzed using gradation analysis. The data were analyzed by student *t*-test or ANOVA and presented as mean ± s.d of three independent experiments.

## Discussion

EndMT, a pathological process that is highly regulated, has already been identified as a critical mechanism for controlling either cardiac development or cardiac fibrosis [4,30]. In adults, abnormal activation of EndMT and differentiation of EndMT-derived fibroblast-like cells to collagen producing myofibroblasts play a significant role in the development and progression of fibrosis in organs such as heart and lung [31–35]. These cells can contribute to the development of cardiac disease, such as fibrosis and low cardiac performance, potentially leading to cardiac arrest and sudden death of the patient [36]. However, very little is known of the molecular and cellular aspects of HCAECs as well as associated factors, and their implication in EndMT and cardiac fibrosis. As a well-studied EndMT inducer, TGF-β plays a key role in EndMT progression [37]. In the present study, we treated HCAECs with different concentrations of TGF-β to verify the inductive effect of TGF-β on EndMT in HCAECs. As expected, HCAECs obtained a spindle-shaped and fibroblast-like morphology and exhibited an EndMT phenotype after TGF-β treatment, especially high-concentration of TGF-β (5 ng/ml). Additionally, the expression trends of EndMT markers, CD31, Vimentin, Snail, Slug, and Twist were significantly changed by TGF-β treatment, showing that TGF-β treatment successfully induces EndMT in HCAECs via promoting Vimentin, Snail, Slug, and Twist expression and hindering CD31 expression. How could we slow down or block this TGF-β-induced EndMT, thus to reduce the myocardial fibrosis damage to the heart?

C-Ski was reported to function as a negative regulator of TGF-β signaling [13,24]. However, the detailed function of c-Ski in TGF-β-induced EndMT in HCAECs was poorly understood. In the present study, our findings show that c-Ski expression was down-regulated by TGF-β in a concentration-dependent manner. In addition, we identified that c-Ski hinders TGF-β-induced EndMT in HCAECs via promoting CD31 expression and hindering Vimentin, Snail, Slug, and Twist expression. However, the potential molecular mechanism of this process still remains to be investigated.

Nowadays, miRNAs have been recognized to be key regulators of various important developmental, homeostatic, and pathogenic pathways. Recent studies propose miRNAs are required for organic development and homeostasis. As exhibited by several independent studies, TGF-β regulates several miRNAs’ expression [38,39]. Here, we used online tools to scan out the candidate miRNAs as regulators of c-Ski, meantime, could be regulated by TGF-β as reported in previous studies. Among the seven candidate miRNAs, miR-155 exhibited the highest inhibitory efficiency on inhibiting c-Ski expression. By directly targeting to c-Ski 3′-UTR, miR-155 negatively regulated c-Ski expression, as confirmed by luciferase reporter gene and Western blot assays. These results inspired us to further validate the detailed role of miR-155 in TGF-β-induced EndMT in HCAEC.

As reported in previous studies, miR-155 could be regulated by TGF-β in intestinal T cells [40]. In addition, miR-155 has been reported to target other genes to regulate epithelial–mesenchymal transition (EMT) process. In hepatocellular carcinoma, miR-155 promotes EMT via targeting P85α and TP53INP1 [41,42]. In murine mammary gland epithelial cells, MiR-155 promotes TGF-β-induced EMT via targeting RhoA [43]. Here, we observed that miR-155 expression was up-regulated by TGF-β treatment in HCAECs, which is consistent with the previous study that TGF-β induces miR-155 in both freshly isolated and LPT lymphoblasts [40]. In addition, we verified that miR-155 participated in the regulation of TGF-β-induced EndMT in HCAECs via promoting Vimentin expression and hindering CD31 expression. Since we have demonstrated that c-Ski participates in TGF-β-induced EndMT, and that miR-155 regulates c-Ski by direct binding to its 3′-UTR in HCAECs, all these data revealed that TGF-β promotes miR-155 expression to regulate c-Ski expression, so as to induce the EndMT in HCAEC.

In conclusion, our results indicate that TGF-β up-regulated the expression of miR-155, down-regulated the expression of c-Ski, and consequently regulated EndMT markers in HCAECs. At the same time, miR-155 targeted c-Ski to regulate the protein expression of EndMT markers CD31, Vimentin Snail, Slug, and Twist. Altogether, the miR-155/c-Ski axis may present a potential therapeutic approach for hindering EndMT in HCAEC so as to contribute to treating cardiac fibrosis.

## Abbreviations

EMT: endothelial–mesenchymal transition
EndMT: epithelial–mesenchymal transition
HCAECs: human coronary artery endothelial cells
TGF-β: transforming growth factor-β
